# Rhodium and Iridium Mediated C-H and O-H Bond Activation of Two Schiff Base Ligands: Synthesis, Characterization and Catalytic Properties of the Organometallic Complexes

**DOI:** 10.3389/fchem.2021.696460

**Published:** 2021-08-09

**Authors:** Poulami Sengupta, Rituparna Das, Papu Dhibar, Piyali Paul, Samaresh Bhattacharya

**Affiliations:** ^1^ Department of Chemistry, Inorganic Chemistry Section, Jadavpur University, Kolkata, India; ^2^ Henkel Limited, Hemel Hempstead, United Kingdom; ^3^ Department of Chemistry and Environment, Heritage Institute of Technology, Kolkata, India

**Keywords:** N-(2′-hydroxyphenyl)furan/thiophene-2-aldimine, C-H and O-H bond activations, organorhodium and organoiridium complexes, formation mechanisms, catalytic C-C cross-coupling reaction

## Abstract

Reaction of [Rh(PPh_3_)_3_Cl] with two Schiff base ligands, *viz*. N-(2′-hydroxyphenyl)furan-2-aldimine (**H**_**2**_ **L** ^**1**^) and N-(2′-hydroxyphenyl)thiophene-2-aldimine (**H**_**2**_ **L** ^**2**^), in refluxing toluene affords organorhodium complexes of type [Rh(PPh_3_)_2_(L)Cl] (L = L^1^ and L^2^). Similar reaction with [Ir(PPh_3_)_3_Cl] yields organoiridium complexes of type [Ir(PPh_3_)_2_(L) (H)] (L = L^1^ and L^2^). Crystal structures of [Rh(PPh_3_)_2_(L^1^)Cl] and [Ir(PPh_3_)_2_(L^2^) (H)] have been determined, where the imine ligands are found to bind to the metal centers as CNO-donors. Structures of [Rh(PPh_3_)_2_(L^2^)Cl] and [Ir(PPh_3_)_2_(L^1^) (H)] have been optimized by density functional theory method. Formation of the organometallic complexes is believed to proceed via C-H and O-H bond activation of the imine ligands. All four complexes show intense absorptions in the visible and ultraviolet regions. Cyclic voltammetry on the complexes shows an oxidation on the positive side of SCE and a reduction on the negative side. The organoiridium complexes are found to efficiently catalyze Suzuki-type C-C cross coupling reactions.

## Introduction

The chemistry of rhodium and iridium has been receiving considerable current attention (Chi et al., 2017; Colombo et al., 2020; Mao et al., 2020; Shaikh et al., 2020; Alsalahi and Trzeciak, 2021; Davison et al., 2021; Monti et al., 2021; Vuuren et al., 2021), largely because of the interesting properties exhibited by the complexes of these metals. As properties of the complexes are dictated primarily by the coordination environment around the metal center, complexation of rhodium by ligands of selected types has been of significant importance, and the present work has originated from our interest in this area (Dutta et al., 2000a; Dutta et al., 2000b; Das et al., 2002; Dutta et al., 2002; Acharyya et al., 2004a; Acharyya et al., 2004b; Acharyya et al., 2005; Acharyya et al., 2006; Basu et al., 2006; Baksi et al., 2007a; Basu et al., 2007; Baksi et al., 2007b; Dasgupta et al., 2008; GuhaRoy et al., 2008; Acharyya et al., 2009; GuhaRoy et al., 2009; Baksi et al., 2010; Basu et al., 2010; Majumder et al., 2011; Seth and Bhattacharya, 2011; Bhattacharya et al., 2012; Paul and Bhattacharya, 2012; Sengupta and Bhattacharya, 2013; Paul et al., 2014). Herein we have selected two Schiff base ligands, *viz*. N-(2′-hydroxyphenyl)furan-2-aldimine (**H**_**2**_ **L** ^**1**^) and N-(2′-hydroxyphenyl)thiophene-2-aldimine (**H**_**2**_ **L** ^**2**^), derived from 2-aminophenol and, furan-2-aldehyde and thiophene-2-aldehyde respectively. The selected ligands may, in principle, bind to metal ions in three possible modes (**I**, **II** and **III**). We were particularly interested to induce binding mode **III** via metal mediated C-H bond activation of these imine ligands. With this target in mind, we selected a rhodium(I) complex [Rh(PPh_3_)_3_Cl], and an analogous iridium(I) complex [Ir(PPh_3_)_3_Cl], as the metal precursors. In our earlier studies we experienced the ability of these two complexes in bringing about C-H bond activation of organic ligands (Dutta et al., 2000b; Dutta et al., 2002; Acharyya et al., 2004b; Acharyya et al., 2005; Basu et al., 2006; Baksi et al., 2007a; Paul and Bhattacharya, 2012; Paul et al., 2014). It is relevant to mention here that metal-mediated C-H bond activation is of significant interest, with particular reference to synthesis of targeted organic molecules (Kumar et al., 2017; Zhu and Zhou, 2017; Cheng et al., 2019; Woźniak et al., 2020; Dongbang et al., 2021; Rej et al., 2021). Reaction of the rhodium and iridium starting materials with the two selected Schiff base ligands has indeed afforded organorhodium and organoiridium complexes. The chemistry of all these complexes is reported here, with particular reference to their formation, structure and catalytic application.

## Experimental

### Materials

Rhodium trichloride and iridium trichloride were purchased from Arora Matthey, Kolkata, India. [Rh(PPh_3_)_3_Cl] and [Ir(PPh_3_)_3_Cl] were prepared by following reported methods (Osborn and Wilkinson, 1967; Acharyya et al., 2004b). Furan-2-carbaldehyde, thiophene-2-carbaldehyde, and 2-aminophenol were obtained from SRL, AVRA Synthesis and Merck, respectively. The two aldimine ligands (**H**_**2**_ **L** ^**1**^ and **H**_**2**_ **L** ^**2**^) were prepared by reacting equimolar mixture 2-aminophenol and the respective aldehyde in hot ethanol. Tetrabutylammonium hexaflurophosphate (TBHP), obtained from Sigma-Aldrich, and AR grade acetonitrile, procured from Merck, India, were used for electrochemical work. All other chemicals and solvents were reagent grade commercial materials and were used as received.

### Syntheses of Complexes

**[Rh**(**PPh**_**3**_**)**_**2**_**(L**^**1**^**)Cl**]: N-(2′-hydroxyphenyl)furan-2-aldimine (20 mg, 0.11 mmol) was dissolved in hot toluene (30 ml) and [Rh(PPh_3_)_3_Cl] (100 mg, 0.11 mmol) was added to it. The reaction mixture was refluxed for 6 h producing a purple solution. The solvent was evaporated under reduced pressure to give a purple solid, which was purified by thin layer chromatography on a silica plate. Using benzene as the eluant a major purple band separated, which was extracted with acetonitrile. Evaporation of this purple extract gave [Rh(PPh_3_)_2_(L^1^)Cl] as a crystalline solid. Yield: 62 mg (68%). Anal. Calc. for C_47_H_37_NO_2_P_2_ClRh: C, 66.56; H, 4.37; N, 1.65. Found: C, 66.18; H, 4.28; N, 1.63%. ^1^H NMR: 5.69 (d, H), 6.17 (t, H, *J* = 3.9 Hz), 6.26 (d, H, *J* = 8.4 Hz), 6.56 (t, H), 6.62 (d, H), 6.99 (d, H), 7.18–7.23 (PPh_3_), 7.64–7.70 (H + PPh_3_)*. IR: 3452, 3057, 2924, 2854, 1744, 1637, 1591, 1533, 1479, 1435, 1403, 1317, 1267, 1188, 1122, 1094, 1030, 999, 841, 746, 694, 538 and 519 cm^−1^.

**[Rh**(**PPh**_**3**_**)**_**2**_**(L**^**2**^**)Cl**]: N-(2′-hydroxyphenyl)thiophene-2-aldimine (22 mg, 0.11 mmol) was dissolved in hot toluene (30 ml) and [Rh(PPh_3_)_3_Cl] (100 mg, 0.11 mmol) was added to it. The reaction mixture was refluxed for 6 h producing a pink solution. The solvent was evaporated under reduced pressure to give a pink solid, which was purified by thin layer chromatography on a silica plate. Using 1:10 acetonitrile-benzene as the eluant a major pink band separated, which was extracted with acetonitrile. Evaporation of this pink extract gave [Rh(PPh_3_)_2_(L^2^)Cl] as a crystalline solid. Yield: 56 mg (60%). Anal. Calc. for C_47_H_37_NOP_2_SClRh: C, 65.32; H, 4.28; N, 1.62. Found: C, 65.29; H, 4.22; N, 1.61%. ^1^H NMR ^1^: 5.68 (d, H, *J* = 3.8 Hz), 6.32 (t, H, *J* = 8.3 Hz), 7.15–7.20 (H + PPh_3_)*, 7.28 (t, H), 7.48 (d, H, *J* = 4.9 Hz), 7.56–7.66 (H + PPh_3_), 7.83 (s, H). IR: 3433, 3055, 2924, 2854, 1661, 1589, 1553, 1464, 1433, 1402, 1315, 1273, 1180, 1121, 1094, 1028, 996, 937, 856, 744, 696, 535 and 517 cm^−1^.

**[Ir**(**PPh**_**3**_**)**_**2**_**(L**^**1**^**) (H)]:** N-(2′-hydroxyphenyl)furan-2-aldimine (19 mg, 0.10 mmol) was dissolved in hot toluene (30 ml) and [Ir(PPh_3_)_3_Cl] (100 mg, 0.10 mmol) was added to it. The reaction mixture was refluxed for 24 h to produce a purple solution. The solvent was evaporated to give a purple solid, which was purified by thin layer chromatography on a silica plate. Using 1:10 hexane-benzene as the eluant a major purple band separated, which was extracted with acetonitrile. Evaporation of this purple extract gave [Ir(PPh_3_)_2_(L^1^) (H)] as a crystalline solid. Yield: 62 mg (70%). Anal. Calc. for C_47_H_38_NO_2_P_2_Ir: C, 62.51; H, 4.21; N, 1.55. Found: C, 62.64; H, 4.23; N, 1.53%. 1H NMR ^1^: -13.76 (t, hydride, *J* = 32.4 Hz), 5.69 (d, H), 6.17 (t, H, *J* = 3.9 Hz), 6.26 (d, H, *J* = 8.4 Hz), 6.56 (t, H), 6.62 (d, H), 6.99 (d, H), 7.18–7.23 (PPh_3_), 7.64–7.70 (H + PPh_3_)*. IR : 3435, 3055, 2924, 2854, 2160, 1747, 1636, 1593, 1542, 1485, 1435, 1385, 1312, 1261, 1178, 1130, 1059, 1032, 998, 836, 746, 696, 538 and 518 cm^−1^.

**[Ir**(**PPh**_**3**_**)**_**2**_**(L**^**2**^**) (H)]:** N-(2′-hydroxyphenyl)thiophene-2-aldimine (20 mg, 0.10 mmol) was dissolved in hot toluene (30 ml) and [Ir(PPh_3_)_3_Cl] (100 mg, 0.10 mmol) was added to it. The reaction mixture was refluxed for 24 h to produce a purple solution. The solvent was evaporated to give a purple solid, which was purified by thin layer chromatography on a silica plate. Using 1:10 hexane-benzene as the eluant a major purple band separated, which was extracted with acetonitrile. Evaporation of this purple extract gave [Ir(PPh_3_)_2_(L^2^) (H)] as a crystalline solid. Yield: 59 mg (65%). Anal. Calc. for C_47_H_38_NOP_2_SIr: C, 61.42; H, 4.14; N, 1.52. Found: C, 61.37; H, 4.16; N, 1.54%. ^1^H NMR: −13.52 (t, hydride, *J* = 34.5 Hz), 5.90 (t, H, *J* = 4.5 Hz), 6.06–6.11 (m, 2H), 6.16 (d, H, *J* = 8.1 Hz), 6.50 (t, H, *J* = 4.7 Hz), 6.64 (d, H, *J* = 7.2 Hz), 7.20–7.33 (PPh_3_), 7.53–7.63 (H + PPh_3_)*. IR: 3462, 3057, 2924, 2854, 2060, 1748, 1635, 1591, 1537, 1479, 1435, 1385, 1308, 1273, 1182, 1094, 1032, 998, 839, 744, 698, 539 and 519 cm^−1^.

### Physical Measurements

Microanalyses (C, H, N) were performed using a Heraeus Carlo Erba 1108 elemental analyzer. Mass spectra were recorded with a Micromass LCT electrospray (Qtof Micro YA263) mass spectrometer. Magnetic susceptibilities were measured using a Sherwood MK-1 balance. IR spectra were obtained on a Perkin-Elmer 783 spectrometer with samples prepared as KBr pellets. Electronic spectra were recorded on a JASCO V-570 spectrophotometer. ^1^H NMR spectra were recorded in CDCl_3_ solution with a Bruker Avance DPX 300 NMR spectrometer using TMS as the internal standard. Electrochemical measurements were made using a CH Instruments model 600A electrochemical analyzer. A platinum disc working electrode, a platinum wire auxiliary electrode and an aqueous saturated calomel reference electrode (SCE) were used in the cyclic voltammetry experiments. All electrochemical experiments were performed under a dinitrogen atmosphere at 298 K. Optimization of ground-state structures and energy calculations for all the complexes were carried out by density functional theory (DFT) method using the Gaussian 09 package (Frisch et al., 2009). GC-MS analyses were performed using a Perkin Elmer CLARUS 680 instrument.

### X-Ray Crystallography

Single crystals of the complex [Rh(PPh_3_)_2_(L^1^)Cl] and [Ir(PPh_3_)_2_(L^2^) (H)] were grown by slow evaporation of an acetonitrile solution of the respective complexes. Selected crystal data and data collection parameters are given in Supplementary Table S1. Data on the crystal were collected on a Bruker SMART CCD diffractometer using graphite monochromated Mo Kα radiation (*λ* = 0.71073 Å). X-ray data reduction, structure solution and refinement were done using SHELXS-97 and SHELXL-97 programs (Sheldrick, 1997). The structures were solved by the direct methods.

### Application as Catalyst

#### General Procedure for Suzuki Coupling Reactions

In a typical run, an oven-dried 10 ml round bottom flask was charged with a known mol percent of catalyst and base, phenylboronic acid (1.2 mmol) and aryl halide (1 mmol) with the appropriate solvents (4 ml). The flask was placed in a preheated oil bath at required temp. After the specified time the flask was removed from the oil bath and water (20 ml) added, followed by extraction with ether (4 × 10 ml). The combined organic layers were washed with water (3 × 10 ml), dried over anhydrous Na_2_SO_4_, and filtered. Solvent was removed under reduced pressure. The residue was dissolved in hexane and analyzed by GCMS.

## Results and Discussion

### Synthesis and Structure

**FIGURE 1:**
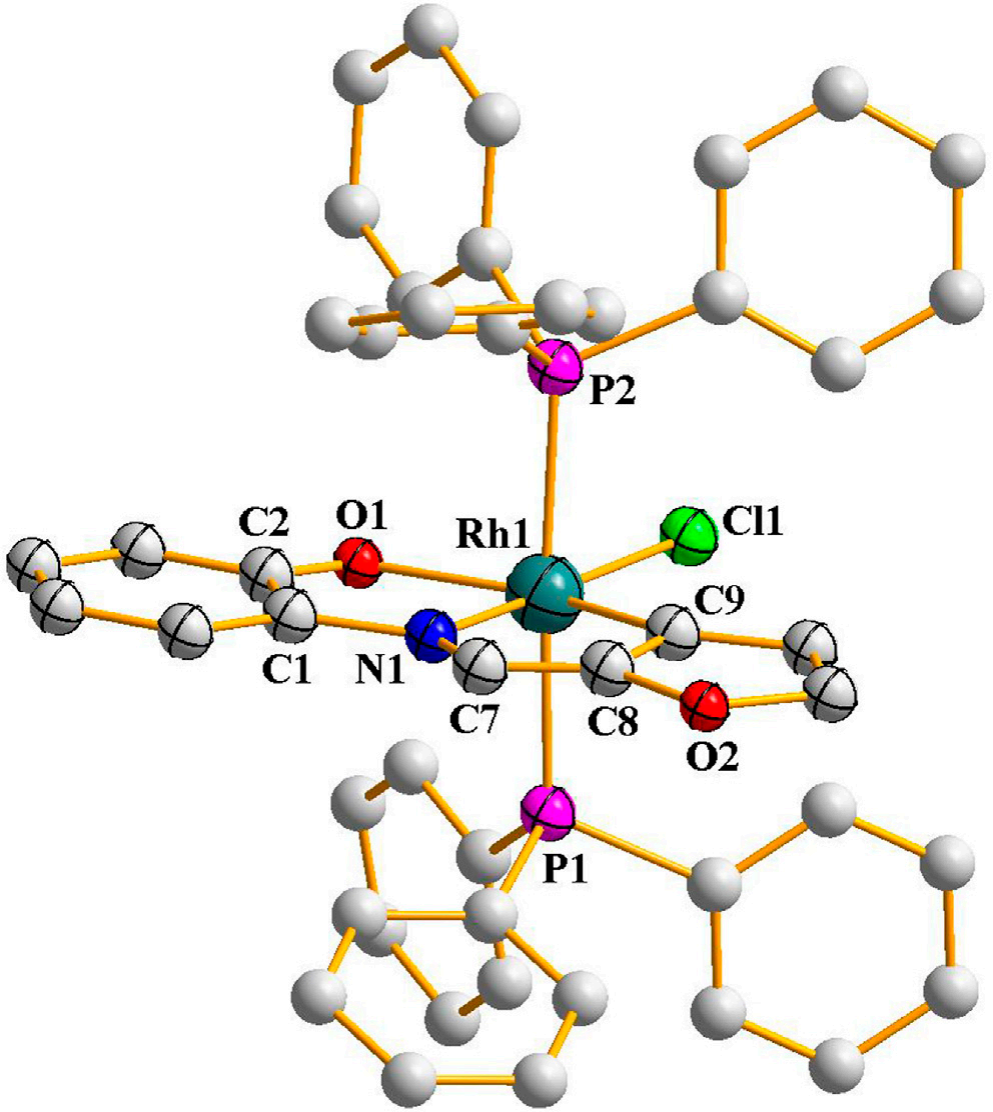
Crystal structure of [Rh(PPh_3_)_2_(L^1^)Cl] (hydrogen atoms are omittedfor clarity).

As delineated in the introduction, the initial goal of the present study has been to synthesize mixed-ligand rhodium and iridium complexes of the two selected ligands, *viz*. N-(2′-hydroxyphenyl)furan-2-aldimine (**H**_**2**_ **L** ^**1**^) and N-(2′-hydroxyphenyl)thiophene-2-aldimine (**H**_**2**_ **L** ^**2**^) and find out their mode of binding to the metal center. We began with reactions of the imine-ligands (**H**_**2**_ **L** ^**1**^ and **H**_**2**_ **L** ^**2**^) with [Rh(PPh_3_)_3_Cl], which proceeded smoothly in refluxing toluene to afford complexes of type [Rh(PPh_3_)_2_(L)Cl] (L = L^1^ and L^2^) in decent yields. Preliminary characterizations (microanalysis, IR, NMR, etc.) on these complexes indicated presence of an imine-ligand, a chloride and two triphenylphosphines in the coordination sphere. In order to find out stereochemistry of these complexes, as well as to ascertain coordination mode of the imine-ligands in them, structure of [Rh(PPh_3_)_2_(L^1^)Cl], was determined by X-ray crystallography. The structure is shown in Figure 1 and some relevant bond parameters are listed in Table 1. The structure revealed that the N-(2′-hydroxyphenyl)furan-2-aldimine ligand is coordinated to rhodium in the tridentate CNO-mode (**III**; M = Rh, X = O), via loss of two protons, *viz*. the hydroxyl proton and a C-H proton from the furan ring. The CNO-coordinated imine ligand, rhodium and chloride constitute one equatorial plane of the octahedron with rhodium at the center, where the chloride is *trans* to the coordinated imine-nitrogen. The two triphenylphosphines have taken up the remaining two axial positions and hence they are mutually *trans*. The CNOP_2_Cl coordination sphere around rhodium is distorted octahedral in nature, which is reflected in all the bond parameters (Table 1) around the metal center. The Rh-C, Rh-N, Rh-O, Rh-P and Rh-Cl distances are all quite normal (Dutta et al., 2000b; Basu et al., 2006; Baksi et al., 2007a). Crystal structure of [Rh(PPh_3_)_2_(L^2^)Cl] could not be determined as good quality crystals of it could not be grown even after repeated attempts. However, its structure was optimized by DFT calculations. The DFT-optimized structure of [Rh(PPh_3_)_2_(L^2^)Cl] (Supplementary Figure S1) is found to be similar to the crystal structure of [Rh(PPh_3_)_2_(L^1^)Cl], and the computed bond parameters (Supplementary Figure S2) are also comparable to those found in [Rh(PPh_3_)_2_(L^1^)Cl]. Besides, we recorded mass spectrum of the [Rh(PPh_3_)_2_(L^2^)Cl] complex, which shows a distinct peak at M/z = 863.99 (Supplementary Figure S2), which corresponds to [M + H]^+^ and is consistent with the composition of this complex.

**Figure:**
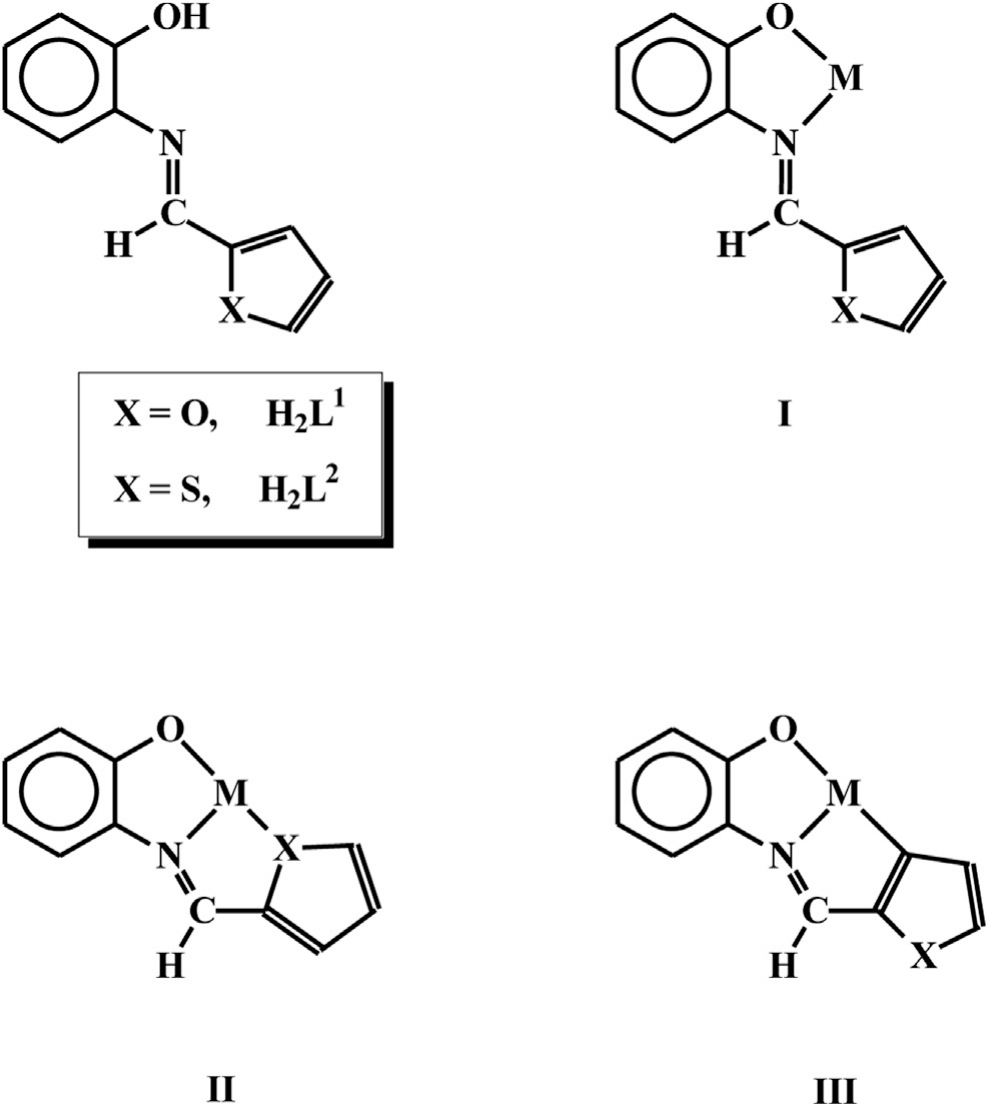


**TABLE 1:** Selected bond distances (Å) and bond angels (°) for [Rh(PPh_3_)_2_(L^1^)Cl].

Bond distances (Ǻ)			
Rh1-Cl1	2.429(15)	C2-O1	1.255(15)
Rh1-P1	2.331(7)	C1-N1	1.47(2)
Rh1-P2	2.377(7)	C7-N1	1.22(3)
Rh1-O1	2.09(2)	C7-C8	1.29(3)
Rh1-N1	1.997(16)	—	—
Rh1-C9	1.96(2)	—	—
**Bond angles (°)**			
P1-Rh1-P2	175.3(3)	O1-Rh1-N1	79.8(6)
N1-Rh1-Cl1	177.4(6)	N1-Rh1-C9	76.8(7)
C9-Rh1-O1	156.5(6)	—	—

**SCHEME 1:**
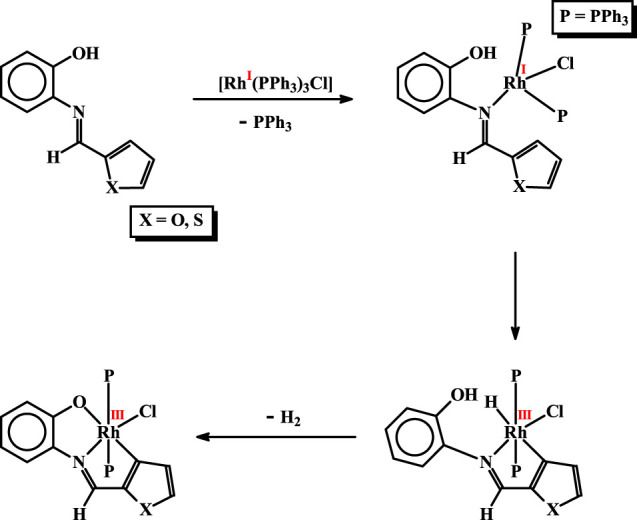
Probable steps behind formation of the rhodium complexes.

Formation of the organorhodium complexes, *viz*. [Rh(PPh_3_)_2_(L^1^)Cl] and [Rh(PPh_3_)_2_(L^2^)Cl], from the reaction of [Rh(PPh_3_)_3_Cl] with the imine ligands (**H**_**2**_ **L** ^**1**^ and **H**_**2**_ **L** ^**2**^), has been quite intriguing. Some speculated sequences behind formation of these complexes, that seem probable, are illustrated in Scheme 1. In the initial step the imine-nitrogen of the **H**_**2**_ **L** ligand seems to bind to the metal center in [Rh(PPh_3_)_3_Cl], via displacement of a PPh_3_, to generate an intermediate. In the next step C-H activation at the 3-position of the heterocyclic ring takes place, whereby oxidative insertion of rhodium into the C-H bond takes place generating a hydrido-rhodium(III) species. Such rhodium(I) assisted C-H bond activation is well documented in the literature (Wiedemann et al., 2006; Gardiner et al., 2015; Chen et al., 2021; Lou et al., 2021; Wang et al., 2021; Yu et al., 2021). The rhodium-bound hydride then interacts with the nearby phenolic O-H fragment, which leads to abstraction of the O-H proton and furnish the final product via elimination of molecular hydrogen. It is interesting to see that in spite of the presence of a recognized donor atom in the heterocyclic ring of the imine ligands, it did not participate in coordination. Instead, C-H activation from the same heterocyclic ring takes place. This is attributable to ability of the rhodium(I) center to activate a proximal C-H bond.

**FIGURE 2:**
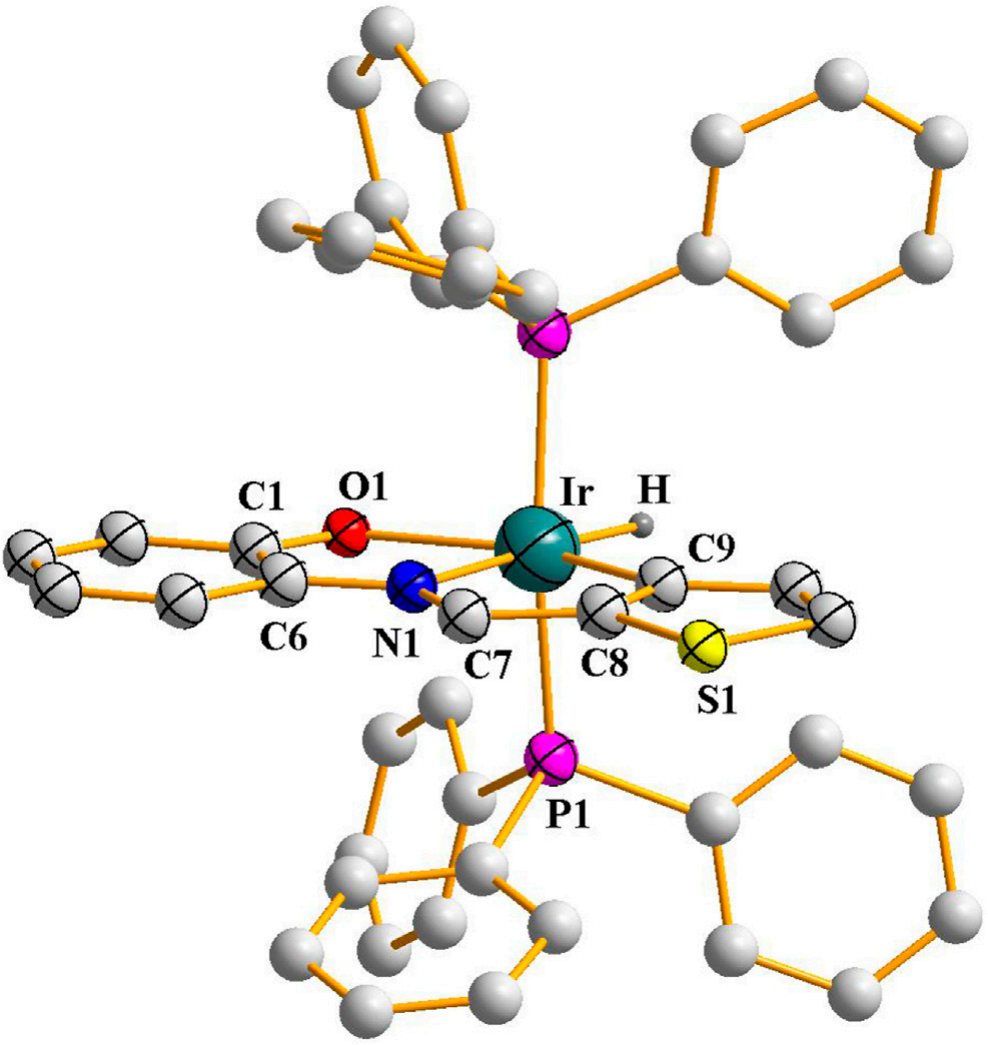
Crystal structure of [Ir(PPh_3_)_2_(L^2^) (H)] (hydrogen atoms, except the hydride hydrogen coordinated to iridium, are omitted for clarity).

Successful synthesis of the organorhodium complexes from the reaction of [Rh(PPh_3_)_3_Cl] with the two selected imine ligands, **H**_**2**_ **L** ^**1**^ and **H**_**2**_ **L** ^**2**^, prompted us to explore analogous iridium reactions. Accordingly, reaction of [Ir(PPh_3_)_3_Cl] was carried out with the imine ligands in refluxing toluene, which afforded complexes of type [Ir(PPh_3_)_2_(L) (H)] (L = L^1^ and L^2^) in good yields. The reflux needed to be continued for a longer period of time for achieving optimum yield of the complexes. Preliminary characterizations (microanalysis, IR, NMR, etc.) on these complexes were found to correspond well with their compositions. Crystal structure of [Ir(PPh_3_)_2_(L^2^) (H)] was determined (Figure 2), which shows that the N-(2′-hydroxyphenyl)thiophene-2-aldimine ligand is bound to iridium in the tridentate CNO-mode (**III**; M = Ir, X = S) via O-H and C-H bond cleavage. Unlike the rhodium complexes, a hydride ligand is found to be coordinated to iridium, and the rest of the structure is qualitatively similar to the that of the rhodium complexes. The Ir-C, Ir-N, Ir-O, Ir-P and Ir-H distances (Table 2) are all quite normal (Acharyya et al., 2004b). Crystal structure determination of [Ir(PPh_3_)_2_(L^1^) (H)] was not possible owing to unavailability of crystals of appropriate quality. Hence its structure was optimized by DFT method, and the DFT-optimized structure (Supplementary Figure S3; Supplementary Table S3) is found to be similar to the crystal structure of [Ir(PPh_3_)_2_(L^2^) (H)]. The mass spectrum of the [Ir(PPh_3_)_2_(L^1^) (H)] complex (Supplementary Figure S4) that shows a distinct peak at M/z = 903.86 consistent with [M + H]^+^ also supports the composition of this complex.

**TABLE 2:** Selected bond distances (Å) and bond angels (°) for [Ir(PPh_3_)_2_(L^2^) (H)].

Bond distances (Ǻ)			
Ir(1)-H(1)	1.65(5)	C1-O1	1.303(9)
Ir(1)-P(1)	2.2994(8)	C6-N1	1.375(18)
Ir(1)-O(1)	2.221(6)	C7-N1	1.271(10)
Ir(1)-N(1)	2.073(5)	C7-C8	1.448(17)
Ir(1)-C(9)	1.990(8)	—	—
**Bond angles (°)**			
P1-Ir-P1a	168.66(3)	O1-Ir-N1	77.1(2)
N1-Ir-H	177.0(3)	N1-Ir-C9	80.0(3)
C9-Ir-O1	157.0(3)	—	—

A formation scheme for the [Ir(PPh_3_)_2_(L) (H)] (L = L^1^ and L^2^) complexes is presented in (Supplementary Scheme S1), which is similar to that for the organorhodium complexes (Scheme 1). The only difference being in the disposition of the coordinated hydride and chloride in the Rh(III) and Ir(III) intermediates, an interesting feature observed earlier by us in similar reactions (Dutta et al., 2000a; Acharyya et al., 2004b; Basu et al., 2006; Baksi et al., 2007a; Paul and Bhattacharya, 2012; Paul et al., 2014), and this is attributable primarily to difference in the experimental conditions. In the last step, elimination of HCl takes place, which is believed to be facilitated by the closeness of Ir-Cl bond to the phenolic O-H fragment in the iridium(III) intermediate, and thus the organoiridium complexes are obtained as end products. It is relevant to mention here that like rhodium(I), iridium(I) is also known to bring about C-H bond activation (Boutry et al., 1997; Tang et al., 2009; Press et al., 2016; Hung et al., 2021; Liu et al., 2021; Slack and Colacot, 2021; Yoshino and Matsunaga, 2021).

### Spectral Properties

Magnetic susceptibility measurements show that all four complexes are diamagnetic, which corresponds to the trivalent state of rhodium and iridium (low-spin d^6^, S = 0) in these complexes. ^1^H NMR spectra of the complexes were recorded in CDCl_3_ solution, which show broad signals within 7.15–7.70 ppm for the coordinated PPh_3_ ligands. The hydride signal in [Ir(PPh_3_)_2_(LL2) (H)] and [Ir(PPh_3_)_2_(L^2^) (H)] complexes is observed respectively at −13.52 ppm and -13.76 ppm as a distinct triplet due to coupling with two equivalent phosphorus nuclei with a coupling constant of ∼19 Hz. Most of the expected aromatic proton signals, as well as the azomethine-proton signal, from the coordinated imine-ligand are clearly observed in the expected region, while a few could not be detected due to their overlap with other signals in the same region.

Infrared spectra of the rhodium and iridium complexes show many bands of different intensities in the 400–4000 cm^−1^ region. No attempt has been made to assign each individual band to a specific vibration. However, comparison with the spectra of the corresponding uncoordinated imine ligands shows that the O-H stretch, observed near 3377 cm^−1^ in the uncoordinated ligands, is absent in the complexes. Three prominent bands, observed near 518, 695 and 745 cm^−1^ in all the complexes, are attributed to the coordinated triphenylphosphine ligands. Several sharp bands are also observed in the [Rh(PPh_3_)_2_(L)Cl] and [Ir(PPh_3_)_2_(L) (H)] (L = L^1^ and L^2^) complexes, which were absent in the [M(PPh_3_)_3_Cl] (M = Rh and Ir), and hence these are attributed to the coordinated imine-ligands. Infrared spectra of [Ir(PPh_3_)_2_(L^1^) (H)] and [Ir(PPh_3_)_2_(L^2^) (H)] show a sharp band at 2161 cm^−1^ and 2060 cm^−1^ respectively, which are attributed to Ir-H stretch.

**FIGURE 3:**
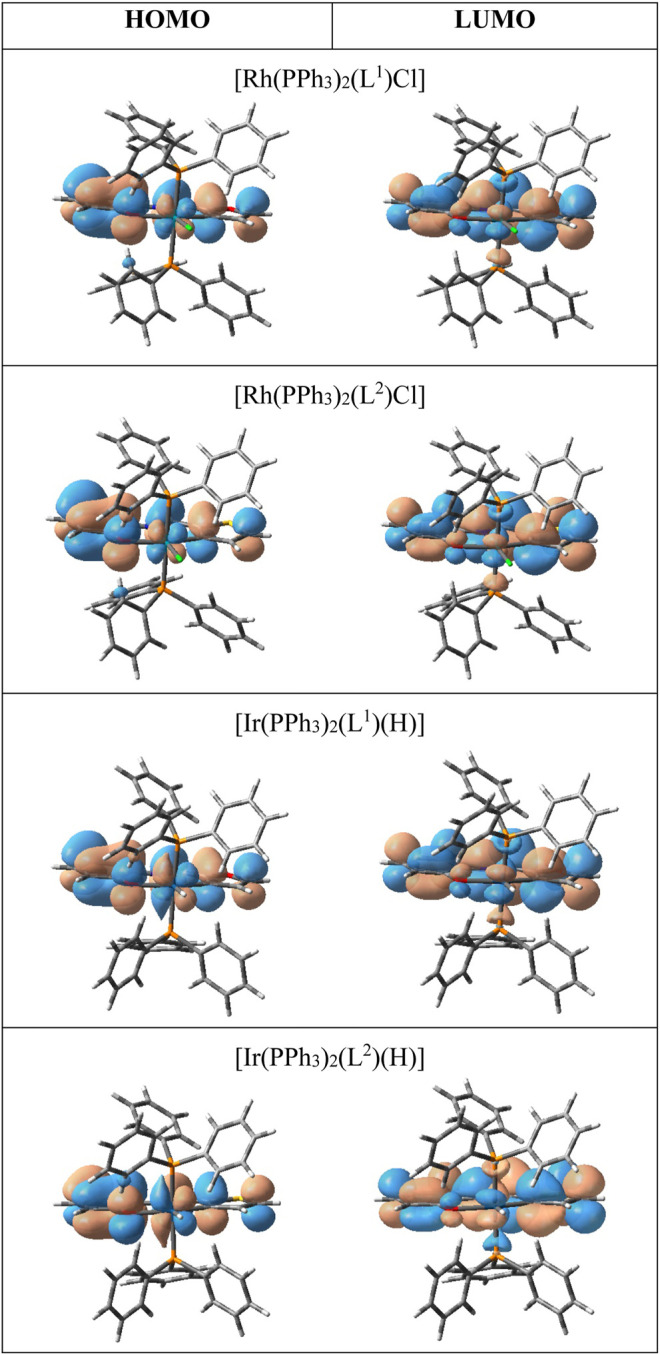
Contour plots of the HOMO and LUMO.

All four complexes are highly soluble in organic solvents like benzene, dichloromethane, acetonitrile, etc., producing intense pink solution. Electronic spectra of the complexes were recorded in dichloromethane solutions. Spectral data are presented in Table 3. The complexes show several intense absorptions in both the visible and ultraviolet regions. The absorptions in the ultraviolet region are attributable to transitions within the ligand orbital. To have an insight into the nature of absorptions in the visible region, DFT calculations were performed on the complexes. Structures of all four complexes were optimized through DFT calculations, and nature of the two Frontier orbitals, *viz*. the highest occupied molecular orbital (HOMO) and the lowest unoccupied molecular orbital (LUMO), was examined. Compositions of the HOMO and LUMO for all four complexes are given in Table 4, and contour plots of these orbitals in the complexes are shown in Figure 3. In all the complexes the HOMO and LUMO are found to be concentrated mostly over the imine-ligand, with much less contribution from the metal center. Hence the lowest energy absorption in the visible region is attributable largely to a transition within these filled and vacant orbitals of the imine ligand.

**TABLE 3:** Electronic spectral and cyclic voltammetric data of the complexes.

Complex	Electronic spectral data^a^ λ_max_, nm (ε, M^−1^cm^−1^)	Cyclic voltammetric data^b^ E/V vs. SCE
[Rh(PPh_3_)_2_(L^1^)Cl]	570(6900), 533(7100), 290(42900)	0.86^c^, −0.25^d^
[Rh(PPh_3_)_2_(L^2^)Cl]	563(5900), 528(5800), 336(11300), 288(23500)	0.97^c^, −0.53^d^
[Ir(PPh_3_)_2_(L^1^) (H)]	538(3800), 308(3600), 266(6100)	0.83^d^, −0.14^e^
[Ir(PPh_3_)_2_(L^2^) (H)]	533(1500), 318(3400), 273(5800)	1.17^d^, −0.43^e^

a In dichloromethane.

b Solvent, acetonitrile; supporting electrolyte, TBHP; scan rate 50 mVs^−1^.

c E_pa_ (anodic peak potential) value.

d E_pc_ (anodic peak potential) value.

**TABLE 4:** Composition of selected molecular orbitals of the complexes.

Complex	Contributing fragments	% Contribution of fragments to	
		HOMO	LUMO
[Rh(PPh_3_)_2_(L^1^)Cl]	Rh	7.83	6.94
	L^1^	92.12	91.91
	Cl	0.05	0.07
	P1	0	0.96
	P2	0	0.12
[Rh(PPh_3_)_2_(L^2^)Cl]	Rh	8.79	9.63
	L^2^	90.31	88.08
	Cl	0.90	0.68
	P1	0	0.72
	P2	0	0.89
[Ir(PPh_3_)_2_(L^1^) (H)]	Ir	8.49	9.57
	L^1^	90.67	87.64
	H	0.84	0.79
	P1	0	1.08
	P2	0	0.92
[Ir(PPh_3_)_2_(L^2^) (H)]	Ir	9.87	10.76
	L^2^	89.11	86.46
	H	1.02	0.98
	P1	0	0.86
	P2	0	0.94

### Electrochemical Properties

Electrochemical properties of the rhodium and iridium complexes were studied by cyclic voltammetry in acetonitrile solution (0.1 M TBHP). Voltammetric data are given in Table 3 and two selected voltammogram are shown in (Supplementary Figure S5). All four complexes show an irreversible oxidative response on the positive side of SCE and an irreversible reductive response on the negative side. Similar cyclic voltammetric responses were observed before for organorhodium and organoiridium complexes of similar type (Dutta et al., 2000a; Acharyya et al., 2004b; Basu et al., 2006; Baksi et al., 2007a; Paul and Bhattacharya, 2012; Paul et al., 2014). In view of composition of the HOMO in all these complexes, the oxidative response is assigned to oxidation of the coordinated imine-ligand. Similarly, based on the composition of the LUMO, the reduction is assigned to reduction of the coordinated imine-ligand.

### Catalytic Properties

Metal center in complexes having ability to undergo two-electron transfer reaction should, in principle, be capable of serving as catalyst for bringing about C-C cross coupling reactions. Palladium complexes are familiar examples of such catalysts (Corbet and Mignani, 2006; Gu et al., 2019; Sebastian and Morales, 2019; Dickmu and Smoliakova, 2020; Lutz and Morandi, 2021). Rhodium and iridium centers in the present group of complexes may also be expected to display such catalytic property, as they can also undergo two-electron transfer reactions. With this expectation we examined the catalytic potential of these rhodium and iridium complexes towards Suzuki type C-C cross-coupling reaction. Initially, coupling reaction between phenylboronic acid and *p*-iodoacetophenone was tried to yield the corresponding biphenyl product. All the experimental parameters were systematically varied to achieve an optimum yield of the product, and after extensive optimization (Supplementary Table S4) it was found that 0.1 mol% [Ir(PPh_3_)_2_(L^2^) (H)] complex as catalyst, Cs_2_CO_3_ (2.4 mmol) as base, polyethylene glycol as solvent, a reaction temperature of 120°C, and a reaction time of 4 h, furnished an excellent (98%) yield of the expected product (entry 1). With the other iridium complex, *viz*. [Ir(PPh_3_)_2_(L^1^) (H)], as catalyst, the product was obtained in slightly lower yield (entry 11). However, the rhodium complexes were found to be much less effective as catalyst, where the product was obtained in poor yields (entries 12 and 13). Hence only the results obtained with [Ir(PPh_3_)_2_(L^2^) (H)] complex as the catalyst precursor are reported here.

The scope of the C-C coupling reaction is summarized in Table 5. Using the optimized the reaction conditions, C-C coupling reaction was performed by varying both the arylhalide and the arylboronic acid. Four different aryliodides and the corresponding four aryl bromides, and two aryl boronic acids (*viz*. phenylboronic acid and *p*-tolylboronic acid) were used in the coupling reactions. From majority of the reactions the expected bi-aryls were obtained in good (76–98%) yields, resulting in an average turn-over number of ∼ 10^3^. Reactions with arylhalides having a–CHO group afforded the biaryls in rather poor (24–27%) yields (entries 4, 8, 12, and 16). These aryl aldehydes were found to get reduced to the corresponding alcohols, which were obtained as the major product. Catalytic transfer hydrogenation of aldehyde seems to have taken place in PEG as the solvent. In similar reactions carried out in the absence of any aryl boronic acid, the alcohols corresponding to the *p*-halobenzaldehydes were obtained as the only product in excellent (≥95%) yields.

**TABLE 5:** Suzuki cross-coupling of aryl halides with aryl boronic acids^*a*^.
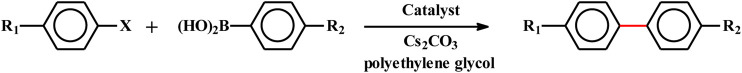

Entry	R_1_	R_2_	X	Yield^b^ (%)
1	COCH_3_	H	I	98
2	H	H	I	95
3	CN	H	I	90
4	CHO	H	I	27^c^
5	COCH_3_	CH_3_	I	97
6	H	CH_3_	I	98
7	CN	CH_3_	I	91
8	CHO	CH_3_	I	24^c^
9	COCH_3_	H	Br	88
10	H	H	Br	89
11	CN	H	Br	78
12	CHO	H	Br	26^c^
13	COCH_3_	CH_3_	Br	86
14	H	CH_3_	Br	84
15	CN	CH_3_	Br	76
16	CHO	CH_3_	Br	26^c^

a Reaction conditions: aryl halide (1.0 mmol), phenylboronic acid (1.2 mmol).

Cs_2_CO_3_ (2.4 mmol), catalyst = [Ir(PPh_3_)_2_(L^2^) (H)] (0.1 mol%), solvent (4 ml).

b Determined by GC-MS on the basis of residual aryl halide.^**4**^.

c In case of -CHO functional group, transfer hydrogenation of–CHO group takes.

Place yielding the corresponding alcoholas the major product. In the absence of.

Phenylboronic acid, 100% formation of -CH_2_OH occurs.

**SCHEME 2:**
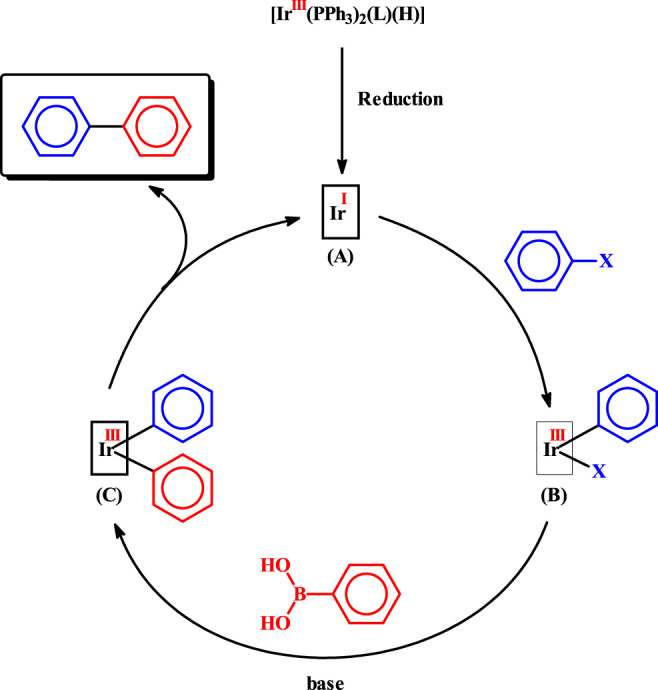
Probable mechanism for the observed C-C coupling reaction.

The observed catalytic C-C coupling reactions are believed to follow the sequences shown in Scheme 2, which are essentially similar to those known for the palladium-catalyzed reactions. In the initial step, the [Ir(PPh_3_)_2_(L) (H)] catalyst-precursor (L = L^1^ or L^2^) undergoes a two-electron reduction reaction to generate an iridium(I) species ^2^, depicted as **A**, and thus entry into the catalytic cycle takes place. The exact nature of **A** is not clear at this moment. However, the soft triphenylphosphines, the hydride, and the nitrogen of the imine-ligand are likely to remain coordinated to the iridium(I) center in **A**. In the following step, oxidative addition of aryl halide takes place generating an iridium(III) species, represented as **B**. Transmetalation happens next, producing another iridium(III) species, depicted as **C**. Finally reductive elimination of the biaryl product takes place, along with simultaneous regeneration of species **A**, and the catalytic cycle thus continues.

Though the observed catalytic efficiency of the iridium complexes is not that great compared to that of the well-known palladium catalysts, it is pleasing to see that iridium complexes are also capable of catalyzing C-C coupling reactions under relatively mild experimental condition. Another noticeable aspect of the observed catalysis is that no additional ligands were necessary for the cross-coupling reactions ^3^. Presence of a soft hydride ligand in the iridium(III) pre-catalyst [Ir(PPh_3_)_2_(L) (H)], might be helpful for its relatively facile reduction to generate the catalytically active iridium(I) species (**A**). Similar reduction of the rhodium(III) complexes [Rh(PPh_3_)_2_(L)Cl], with a chloride instead of the hydride, was presumably not so favorable. And this difference in composition of the pre-catalysts accounts for the observed difference in catalytic efficiency of the iridium and rhodium complexes.

## Conclusion

The present study shows that the two selected imine-ligands, **H**_**2**_ **L** ^**1**^ and **H**_**2**_ **L** ^**2**^, can readily undergo rhodium and iridium mediated activation of the C-H and O-H bonds producing organometallic complexes. Interestingly, the available donor atom X (X = O or S) in the heterocyclic ring of the imine-ligands is found to remain unreactive, whereas a C-H bond in the same ring underwent cleavage. The iridium complexes serve as efficient catalyst precursor for Suzuki type about C-C cross coupling reactions. It is worth mentioning in this context that though iridium complexes are known to serve as photocatalyst for C-C coupling reactions of some other types (Donabauer and Konig, 2021), iridium-catalyzed Suzuki type C-C coupling appears to be unprecedented.

## Data Availability

The original contributions presented in the study are included in the article/Supplementary Material, further inquiries can be directed to the corresponding author.

## Footnotes

1 Chemical shifts for all NMR data are given in ppm and the multiplicity of the signals, along with the associated coupling constant(s), is given in parentheses. Overlapping signals are marked with an asterisk (*).

2 The solvent and/or base served as the reducing agent.

3 Attempted reactions with additional phosphine ligands did not have any positive effect on the catalytic reactions.
