# Supplementary material for: Rhodium and Iridium Mediated C-H and O-H Bond Activation of Two Schiff Base Ligands: Synthesis, Characterization and Catalytic Properties of the Organometallic Complexes
Source: Front Chem. 2021 Aug 9;9:696460. doi: 10.3389/fchem.2021.696460 (PMC8380818; doi:10.3389/fchem.2021.696460)
Supplement: Supplementary file 1 [file DataSheet1.docx]

**Supplementary material**

**Rhodium and iridium mediated C-H and O-H bond activation of two Schiff base ligands: Synthesis, characterization and catalytic properties of the organometallic complexes**

**Poulami Sengupta,^a,b^ Rituparna Das,^a‡^ Papu Dhibar,^a^ Piyali Paul,^a,c^ and Samaresh Bhattacharya^a^***

^a^*Department of Chemistry, Inorganic Chemistry Section, Jadavpur University,*

*Kolkata 700 032, India*

^b^ *Present Address: Henkel Limited, Wood Lane End, Hemel Hempstead, HP2 4RQ, UK*

^c^ *Present Address: Department of Chemistry and Environment, Heritage Institute of Technology, Kolkata 700 107, India*

* Corresponding author: E-mail: [samaresh_b@yahoo.com](mailto:samaresh_b@yahoo.com)

**^‡^** Contributed as M.Sc. Project Fellow from the Department of Chemistry,

Jadavpur University, Kolkata 700032

**Table S1.** Crystallographic data for [Rh(PPh_3_)_2_(L^1^)Cl] and [Ir(PPh_3_)_2_(L^2^)(H)]

| Empirical formula | C_47_H_37_NO_2_P_2_ClRh | C_47_H_38_NOP_2_SIr |
| --- | --- | --- |
| Formula weight | 847.48 | 918.99 |
| Crystal system | Monoclinic | Monoclinic |
| Space group | Ia | C2/c |
| *a* /Å | 20.8815(8) | 23.8049 (9) |
| *b* /Å | 8.8149(3) | 8.7872 (3) |
| *c* /Å | 23.7955(7) | 20.6147 (7) |
| *β* /° | 115.885(4) | 115.097 (3) |
| *V* /Å^3^ | 3940.6(3) | 3905.0 (3) |
| *Z* | 4 | 4 |
| *λ* /Å | 0.71073 | 0.71073 |
| D_calcd_/mg m^-3^ | 1.429 | 1.565 |
| *F*(000) | 1736 | 1836 |
| Crystal size /mm^3^ | 0.22 × 0.22 × 0.24 | 0.15 × 0.25 × 0.35 |
| *T* /K | 298 | 298 |
| *μ* /mm^-1^ | 0.623 | 3.592 |
| Collected reflections | 14180 | 34073 |
| *R*_int_ | 0.042 | 0.035 |
| Independent reflections | 3666 | 5572 |
| *R*1^a^ | 0.1221 | 0.0275 |
| *wR*2^b^ | 0.3693 | 0.0705 |
| GOF^c^ | 1.57 | 1.03 |

*^a^* R1 = Σ⎥⎥ F_o_⎥ -⎥ F_c_⎥⎥ / Σ ⎥ F_o_⎥.

*^b^* wR2= [ Σ {w(F_o_^2^-F_c_^2^)^2^} / Σ{w(F_o_^2^)}]^1/2^.

*^c^* GOF = [Σ(w(F_o_^2^-F_c_^2^)^2^)/(M-N)]^1/ 2^, where M is the number of reflections and N is the number of parameters refined.

**
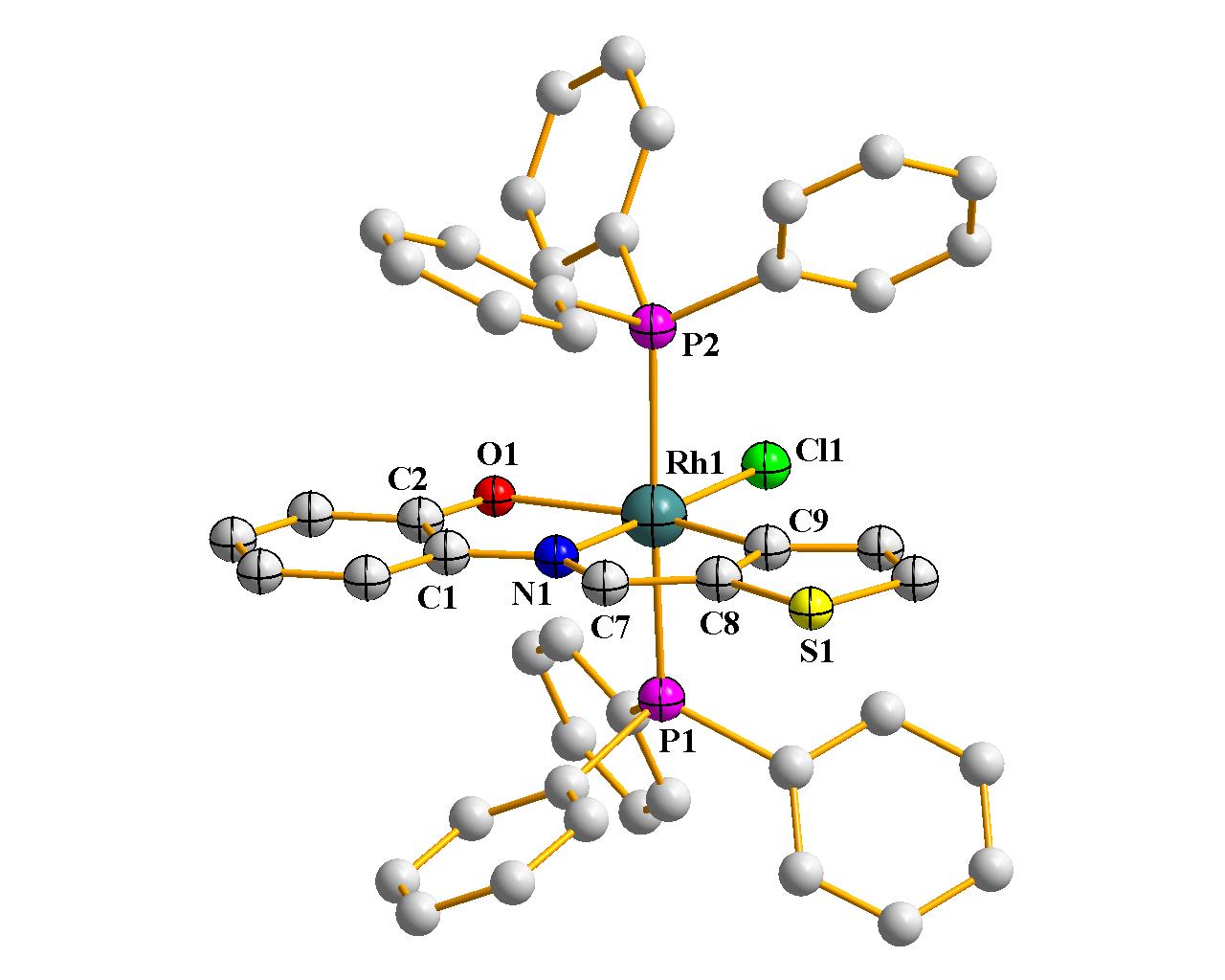
**

**Fig. S1.** DFT-optimized structure of [Rh(PPh_3_)_2_(L^2^)Cl]

(hydrogen atoms are omitted for clarity).

**Table S2.** Some computed bond distances (Å) and bond angels (°) for the

DFT-optimized structure of [Rh(PPh_3_)_2_(L^2^)Cl]

| Bond distances (Ǻ) | | | |
| --- | --- | --- | --- |
| Rh1-Cl1 | 2.4417 | C2-O1 | 1.3130 |
| Rh1-P1 | 2.4525 | C1-N1 | 1.4030 |
| Rh1-P2 | 2.4527 | C7-N1 | 1.3082 |
| Rh1-O1 | 2.2202 | C7-C8 | 1.4283 |
| Rh1-N1 | 2.0403 |  |  |
| Rh1-C9 | 2.0321 |  |  |
| Bond angles (°) | | | |
| P1-Rh1-P2 | 174.090 | O1-Rh1-N1 | 78.569 |
| N1-Rh1-Cl1 | 178.200 | N1-Rh1-C9 | 81.511 |
| C9-Rh1-O1 | 160.079 |  |  |


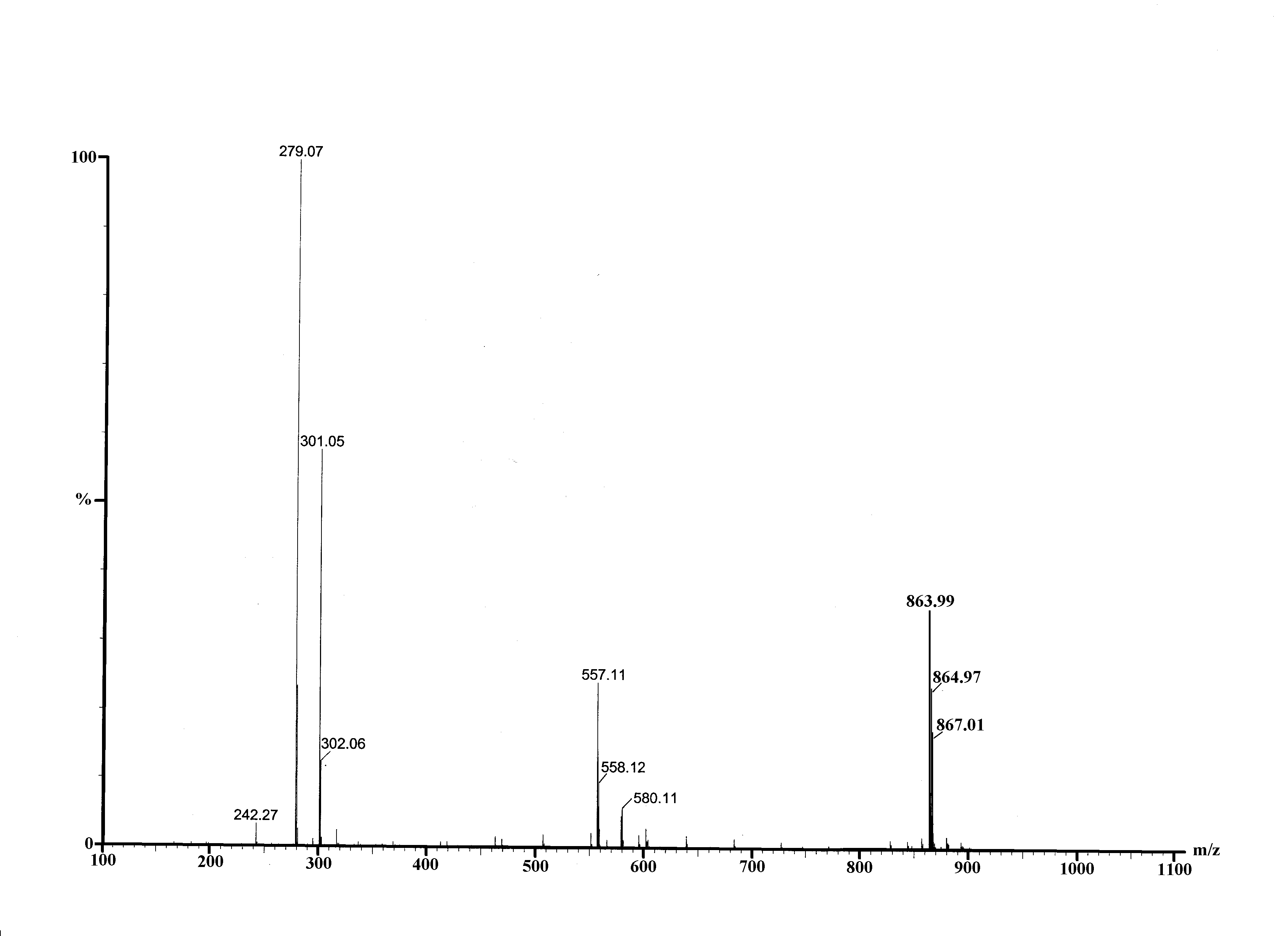


**Fig. S2.** Mass spectrum of [Rh(PPh_3_)_2_(L^2^)Cl].


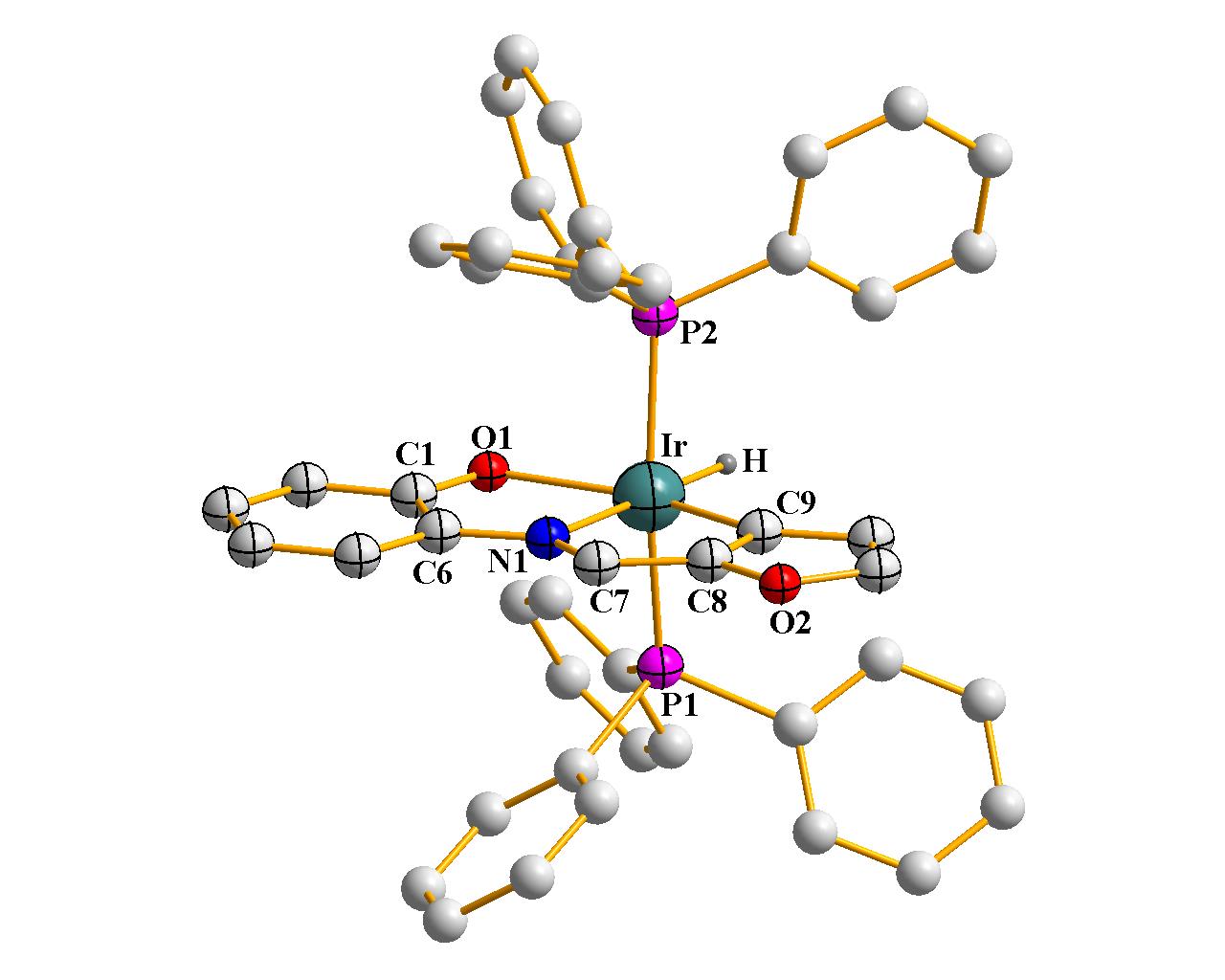


**Fig. S3.** DFT-optimized structure of [Ir(PPh_3_)_2_(L^1^)(H)] (hydrogen atoms, except the hydride hydrogen coordinated to iridium, are omitted for clarity).

**Table S3.** Some computed bond distances (Å) and bond angels (°) for the

DFT-optimized structure of [Ir(PPh_3_)_2_(L^1^)(H)].

| Bond distances (Ǻ) | | | |
| --- | --- | --- | --- |
| Ir-H | 1.6263 | C1-O1 | 1.3206 |
| Ir-P1 | 2.3677 | C6-N1 | 1.4006 |
| Ir-P2 | 2.3571 | C7-N1 | 1.3088 |
| Ir-O1 | 2.2087 | C7-C8 | 1.4238 |
| Ir-N1 | 2.1315 |  |  |
| Ir-C9 | 2.0473 |  |  |
| Bond angles (°) | | | |
| P1-Ir-P2 | 166.782 | O1-Ir-N1 | 77.337 |
| N1-Ir-H | 179.668 | N1-Ir-C9 | 79.794 |
| C9-Ir-O1 | 157.080 |  |  |


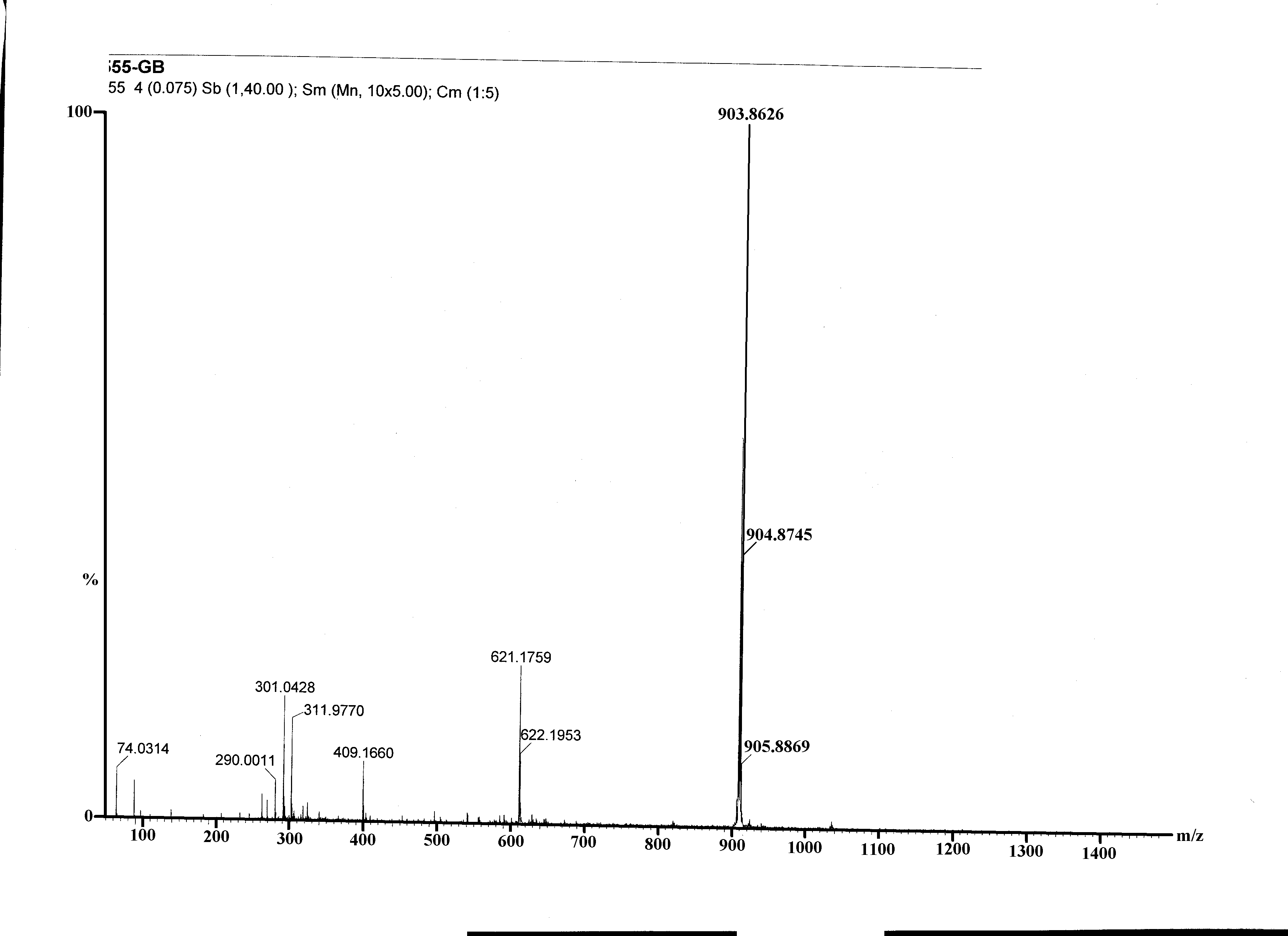


**Fig. S4.** Mass spectrum of [Ir(PPh_3_)_2_(L^1^)(H)].

**Scheme S1.** Probable steps behind formation of the iridium complexes.

| 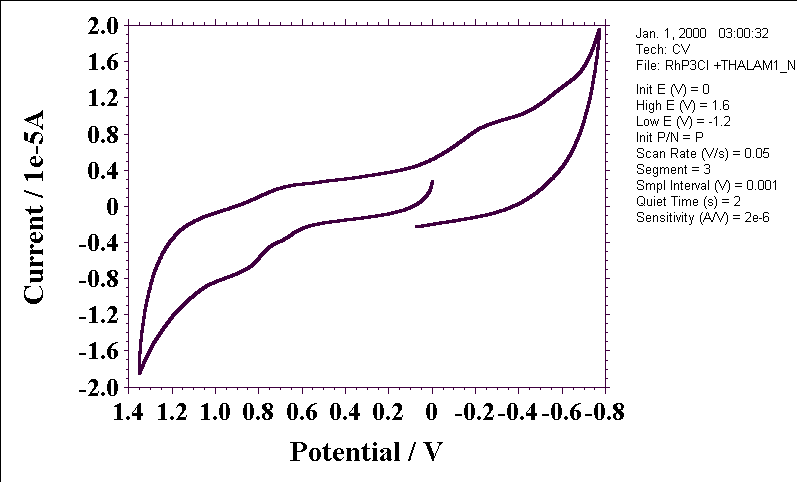 |
| --- |
| 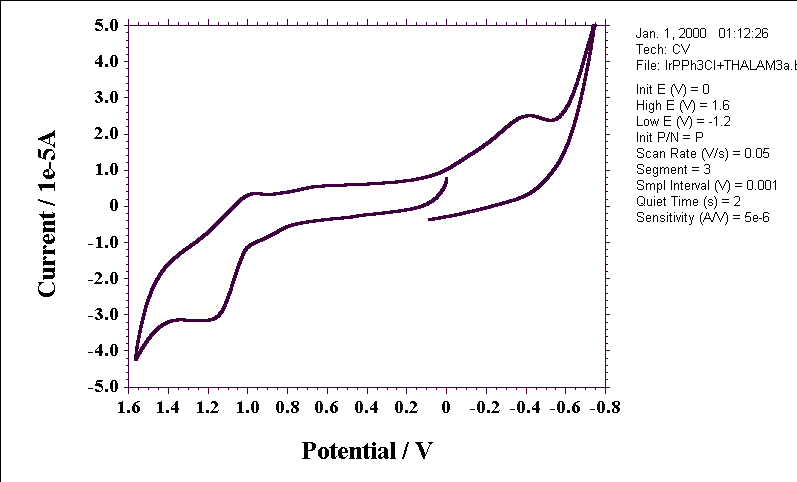 |

**Fig. S5.** Cyclic voltammogram of [Rh(PPh_3_)_2_(L^1^)Cl] (top) and [Ir(PPh_3_)_2_(L^2^)(H)] (bottom) in acetonitrile solution (0.1 M TBHP) at a scan rate of 50 mV s^-1^

**Table S4.** Optimization of experimental parameters for Suzuki type

C-C cross-coupling reaction.*^a^*

| Entry | Catalyst*^b^* | mol % of catalyst | Solvent | Base | Temp, ºC | Time,  h | Yeild*^c^*,  % |
| --- | --- | --- | --- | --- | --- | --- | --- |
| 1 | **Ir-2** | 0.1 | PEG | Cs_2_CO_3_ | 120 | 4 | 98 |
| 2 | **Ir-2** | 0.05 | PEG | Cs_2_CO_3_ | 120 | 4 | 53 |
| 3 | **Ir-2** | 0.1 | PEG | Cs_2_CO_3_ | 100 | 4 | 71 |
| 4 | **Ir-2** | 0.1 | ethanol | Cs_2_CO_3_ | 78.6 | 4 | 42 |
| 5 | **Ir-2** | 0.1 | 2-propanol | Cs_2_CO_3_ | 82.5 | 4 | 48 |
| 6 | **Ir-2** | 0.1 | PEG | Cs_2_CO_3_ | 120 | 3 | 79 |
| 7 | **Ir-2** | 0.1 | PEG | Cs_2_CO_3_ | 120 | 5 | 98 |
| 8 | **Ir-2** | 0.1 | PEG | ----- | 120 | 4 | 0 |
| 9 | **Ir-2** | 0.1 | PEG | Na_2_CO_3_ | 120 | 4 | 65 |
| 10 | **Ir-2** | 0.1 | PEG | K_2_CO_3_ | 120 | 4 | 69 |
| 11 | **Ir-1** | 0.1 | PEG | Cs_2_CO_3_ | 120 | 4 | 95 |
| 12 | **Rh-1** | 0.1 | PEG | Cs_2_CO_3_ | 120 | 4 | 23 |
| 13 | **Rh-2** | 0.1 | PEG | Cs_2_CO_3_ | 120 | 4 | 27 |

*^a^* Reaction conditions: aryl halide (1.0 mmol), phenylboronic acid (1.2 mmol),

base (2.4 mmol), solvent (4 ml).

*^b^* **Ir-1** = [Ir(PPh_3_)_2_(L^1^)(H)]; **Ir-2** = [Ir(PPh_3_)_2_(L^2^)(H)]; **Rh-1** = [Rh(PPh_3_)_2_(L^1^)Cl];

**Rh-2** = [Rh(PPh_3_)_2_(L^2^)Cl].

*^c^* Determined by GCMS.
